# Vanadium Phosphorus Oxide/Siliceous Mesostructured Cellular Foams: efficient and selective for sustainable acrylic acid production via condensation route

**DOI:** 10.1038/s41598-019-53180-8

**Published:** 2019-11-18

**Authors:** Jun Liu, Peiwen Xu, Pengcheng Wang, Zhijia Xu, Xinzhen Feng, Weijie Ji, Chak-Tong Au

**Affiliations:** 1 Key Laboratory of Mesoscopic Chemistry, MOE, School of Chemistry and Chemical Engineering, Nanjing University, Nanjing, 210023 China; 2 Department of Chemistry, Hong Kong Baptist University, Kowloon Tong, Hong Kong

**Keywords:** Catalysis, Chemical synthesis

## Abstract

A new type of supported vanadium phosphorus oxide (VPO) with self-phase regulation was simply fabricated (organic solvent free) for the first time by depositing the specific VPO precursor NH_4_(VO_2_)HPO_4_ onto the Siliceous Mesostructured Cellular Foams (MCF) with controlled activation. The resulting materials were found to be highly efficient and selective for sustainable acrylic acid (AA) plus methyl acrylate (MA) production via a condensation route between acetic acid (HAc) and formaldehyde (HCHO). A (AA + MA) yield of 83.7% (HCHO input-based) or a (AA + MA) selectivity of 81.7% (converted HAc-based) are achievable at 360 °C. The systematic characterizations and evaluations demonstrate a unique surface regulation occurring between the MCF and the NH_4_(VO_2_)HPO_4_ precursor. NH_3_ release upon activation of NH_4_(VO_2_)HPO_4_ precursor together with adsorption of NH_3_ by MCF automatically induces partial reduction of V^5+^ whose content is fine-tunable by the VPO loading. Such a functionalization simultaneously modifies phase constitution and surface acidity/basicity of catalyst, hence readily controls catalytic performance.

## Introduction

Acrylic acid (AA) together with methyl acrylate (MA) were widely applied for producing adhesives, plastics, PAN fibers, hydrogel, biomimetic materials, coatings etc.^1–3^. At present, the traditional way to make AA involves successive oxidation of propylene^4–9^. The propylene feedstock, however, is closely associated with the oil reserve and thought to be non-sustainable, hence an enthusiasm raised by the volatile of crude oil price to explore alternative production route of AA through sustainable resources becomes stronger^10–12^. In recent years, López Nieto and co-workers systematically studied the oxidehydration of glycerol to AA through a one-pot process^13–15^. Furthermore, AA manufacture through the condensation between HCHO and HAc is also considered as a promising approach. Currently, both HCHO and HAc are made from methanol, whereas HAc is largely produced via catalytic carbonylation of methanol. On the other hand, HCHO is fabricated via methanol oxidation. At present, methanol is mainly originated from natural gas/coal, and largely distributed in Asian and North America. As a result, both HAc and HCHO are getting low-priced. Due to an obvious over-production capacity of HAc, it is highly desirable to explore a new process transforming HAc to the value-added chemical such as AA. Researchers have devoted their efforts to the condensation route employing HAc and HCHO^16–19^.

Typically, the gas phase condensation using HCHO and HAc can be catalyzed by solid acids and/or bases, or acid-base bi-functional catalysts^20–22^. Among the basic catalysts, a variety of cesium supported catalysts, using SiO_2_ and SBA-15 as supports, were investigated for the objective reaction^22,23^. Real practice of this kind of catalyst, however, was technically difficult since the AA/MA yield is not high enough and the alkali metal oxides are sensitive to atmosphere. Ai *et al*. found out that the acid-base bi-functional catalyst V_2_O_5_-P_2_O_5_ outperformed the acidic counterparts^18^.

The vanadium phosphorus oxide (VPO) type catalyst is versatile for selective oxidation of light alkanes such as *n*-butane and propane^24,25^. In recent years, people paid much attention to the condensation route over the VPO type catalyst. Yang *et al*.^26^ reported that the relative V/P content was curial for catalytic behavior. Ji and co-workers^20^ observed a VPO catalyst containing a great amount of δ-VOPO_4_ species efficient for the condensation route. Furthermore, Wang *et al*.^27^ found that the V^4+^ and V^5+^ species in the (VO)_2_P_2_O_7_ and VOPO_4_ phase respectively gives the critical V^4+^ to V^5+^ ratio influential on catalytic activity of the VPO type catalyst. The complexity in phase composition, oxidation state of V, and surface atomic ratio P to V of VPO catalyst requires a deeper understanding the structure-performance correlation for the target reaction.

So far, a few supported VPO systems have been reported for the target reaction^28–31^. Hu *et al*.^31^ reported a series of supported VPOs on SiO_2_, SBA-15, and HZSM-5, and found that the kind of support has a substantial impact on the acid-base property of catalyst, showing distinct catalytic behavior as a result. Zhao *et al*.^28^ investigated the condensation reaction over the VPO supported on TiO_2_, SiO_2_, Sb_2_O_3_, γ-Al_2_O_3_, ZrO_2_, Nb_2_O_5_, and ƞ-Al_2_O_3_, and found out that the support type would significantly modify the structure and composition of active phase as well as the acid-base characteristics.

Three-dimensional (3D) cellular solid foams have been widely used in the field of materials engineering such as electrochemical porous electrodes^32,33^. Siliceous Mesostructured Cellular Foams (MCF), with well-defined uniform ultra-large mesopores and surface area as well as aerogel-like 3D pore channels were utilized in various applications such as catalysis, separation, and adsorption^34^. Different from bulk SiO_2_ or porous materials with 2D pore channels (MCM-41 and SBA-15), MCF is a new class of 3D hydrothermally robust materials with ultra-large mesopores (up to 50 nm). In terms of the textural and framework structure, the MCF materials resemble aerogels and comprise uniform spherical cells interconnected by windows with a narrow size distribution. These novel pore characteristics of MCF provide more open porous networks, allowing accommodation of more guest species (such as VPO herein), and more accessible contact between reactants and active sites and thus improved diffusion ability^35,36^. In this study, the choice of MCF as support was not limited to these benefits, the MCF can function the NH_3_-assisted V^5+^ reduction and vary the phase constitution and consequently the surface V^4+^/V^5+^ ratio, which in turn simultaneously modifies the surface acidity of catalyst and hence controls the reaction performance. Therefore, a new type of supported VPO catalyst can be developed with a feature of self-regulation of active phase and surface acidity by employing NH_4_(VO_2_)HPO_4_ as the VPO precursor and systematically varying its loading on MCF support. The quantity of released NH_3_ and the degree of V^5+^ reduction and thus the surface ratio of V^4+^/V^5+^ together with the corresponding catalyst surface acidity can be fine-tuned in sample serial. In such a way, catalyst nature and activity can be systematically controlled. Upon the characterizations using BET, XRD, XPS, NH_3_-TPD, plus Pyridine-IR, detailed structure-performance relationship can be established.

In most of previous studies on the target reaction, the performance was evaluated in terms of the HCHO-based yield. Though it is useful, while insufficient to reveal the overall reaction process, especially in view of the practical application. This is because HAc is commonly excess to HCHO in great amount, and the by-products such as acetone and CO_x_ are largely originated from HAc. In this study, we calculated the HAc-based (AA + MA) selectivity for the first time as well as the overall carbon balance which is essential to get a complete picture of the reaction but did not appear in the early literature.

## Results and Discussion

### XRD analysis

**Figure 1:**
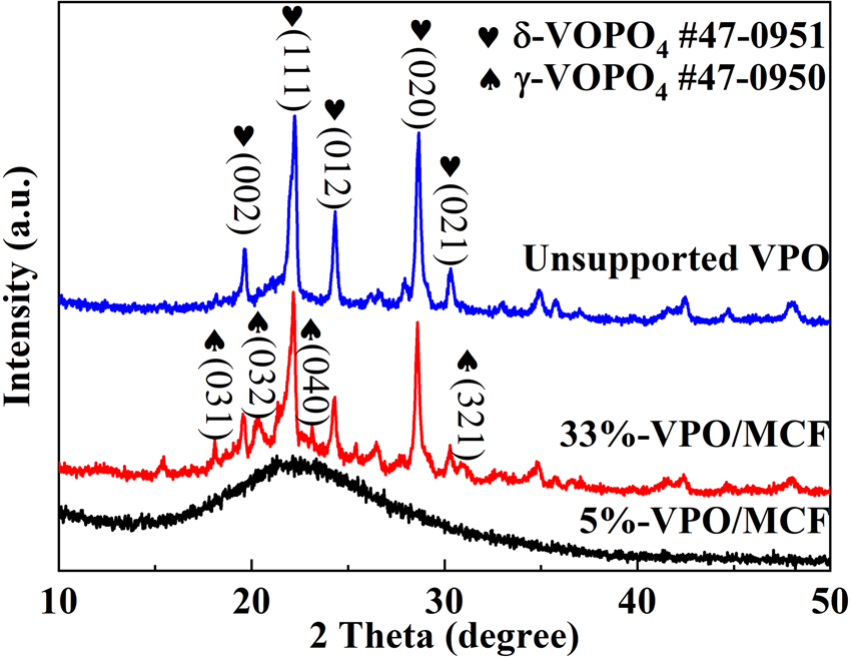
XRD patterns of the representative catalysts.

The XRD patterns of various catalysts were displayed in Fig. 1. The supports could largely affect the structure of supported catalysts^28^. All the diffraction lines can be ascribed to the δ-VOPO_4_ phase (PDF#47-0951) and no other peak was observed. It means the V, P-containing precursor (NH_4_(VO_2_)HPO_4_) was transformed to nearly pure δ-VOPO_4_ entity under the employed activation conditions. Notably, the phase structure was obviously changed after activation when this VPO precursor was deposited on MCF. The XRD pattern of 5%-VPO/MCF shows only one broad peak centered at 2θ = 15–35°, characteristic of siliceous materials^22^. The VPO entity of low loading is undetectable on the MCF surface. It is either in amorphous state or well-dispersed and difficult to be distinguished by XRD. As the VPO loading is raised to 33%, all the diffraction lines can be assigned to the δ-VOPO_4_ (PDF#47-0951) and γ-VOPO_4_ (PDF#47-0950) phases. Compared to the unsupported VPO sample, a VPO/MCF sample comprises binary VOPO_4_ phases after thermal activation of dispersed NH_4_(VO_2_)HPO_4_ precursor on MCF [NH_4_(VO_2_)HPO_4_ → VOPO_4_ + NH_3_ + H_2_O]. Interestingly, according to our previous study^37^, a binary-phase VPO catalyst consisting of δ-VOPO_4_ and γ-VOPO_4_ exhibited promising catalytic performance. Therefore, the 33%-VPO/MCF with the spontaneously generated δ-VOPO_4_ and γ-VOPO_4_ phases would be highly favorable owing to the unique inter-phase conjunction for the reaction^37^.

### Raman analysis

**Figure 2:**
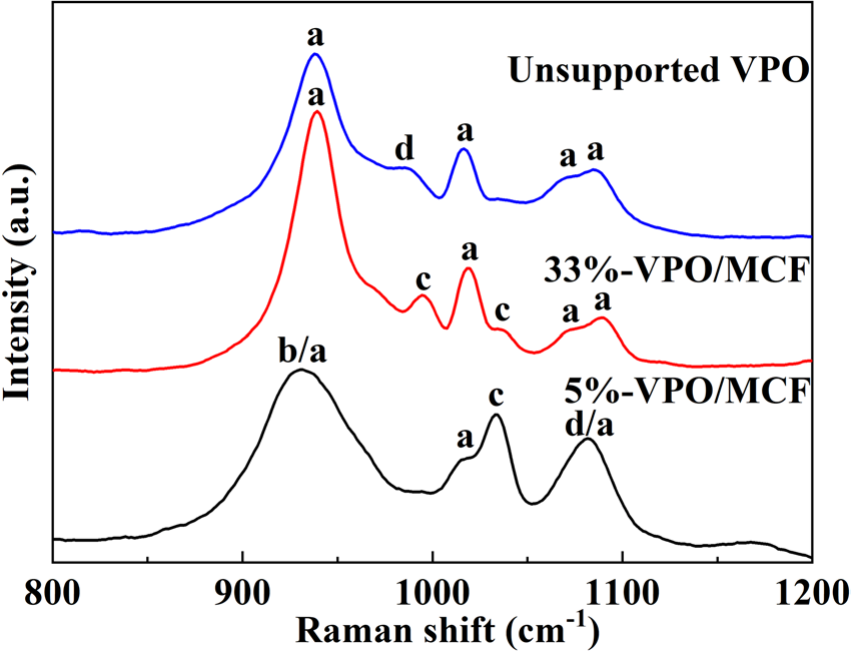
Raman spectra of the catalysts, a: δ-VOPO_4_, b: (VO)_2_P_2_O_7_, c: γ-VOPO_4_, and d: β-VOPO_4_.

Figure 2 exhibits the Raman spectra of various catalysts. Two distinct spectroscopic regions can be classified for various VOPO_4_ species^38^, namely, the range of 850–1200 cm^−1^ corresponding to the P-O and V-O stretching modes. According to the previous reports^38,39^, the intensive band of ca. 936 cm^−1^ together with the weak bands at 1020, 1075, and 1090 cm^−1^ are characteristic of δ-VOPO_4_ species for the unsupported VPO catalyst, and the weak band of 986 cm^−1^ is due to β-VOPO_4_. Clearly, the unsupported VPO contains most of δ-VOPO_4_ plus minor β-VOPO_4_ phases. Surprisingly, as shown in Fig. 2, the VPO entities in 5%-VPO/MCF are found to be (VO)_2_P_2_O_7_, γ-VOPO_4_, and β-VOPO_4_, whereas the δ-VOPO_4_ is considerably reduced. The dominant δ-VOPO_4_ together with minor γ-VOPO_4_ is confirmed in 33%-VPO/MCF, consistent with the corresponding XRD observation. In terms of the XRD and Raman measurements, one can figure out that the pure catalyst precursor NH_4_(VO_2_)HPO_4_ is essentially transformed into δ-VOPO_4_ upon activation. In a low loading sample such as 5%-VPO/MCF, (VO)_2_P_2_O_7_, γ-VOPO_4_, and β-VOPO_4_ plus remarkably reduced δ-VOPO_4_ co-exist; whereas in a high loading sample such as 33%-VPO/MCF, dominant δ-VOPO_4_ plus minor γ-VOPO_4_ co-exist. Obviously, significant change in VPO phase composition would result in notable variation in I_V_^4+^/I_V_^5+^ (intensity ratio of V^4+^ and V^5+^ species). According to literature^20^, a VPO catalyst with a suitable I_V_^4+^/I_V_^5+^ value would outperform the one with simplex valence state.

### XPS analysis

**Figure 3:**
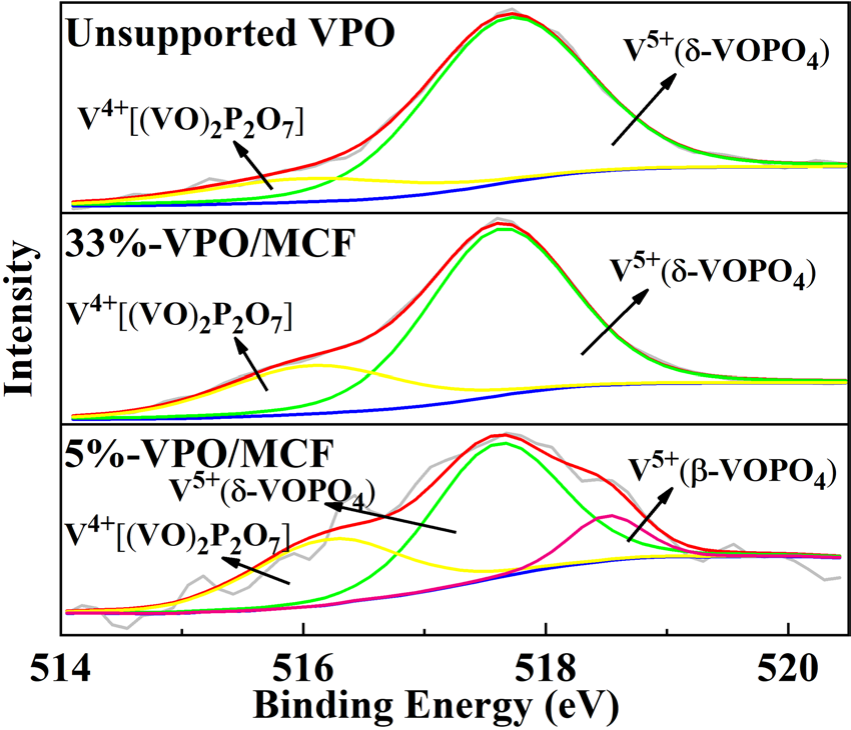
Curve-fitting analysis of the V2p_3/2_ peaks of the catalysts.

The XPS analysis tells the surface elemental composition on the different catalysts. The binding energies were calibrated against 284.6 eV^40^. The spectra of unsupported VPO and 33%-VPO/MCF (Fig. 3) can be deconvoluted into two separate peaks centered at 517.6 and 516.0 eV, corresponding to the signal of V^5+^ 2p_3/2_ and V^4+^ 2p_3/2_, respectively^41,42^. The spectrum of 5%-VPO/MCF can be deconvoluted into three components centered at 518.7, 517.6, and 516.0 eV, corresponding to the signal of V^5+^ 2p_3/2_ associated with β-VOPO_4_ and δ-VOPO_4_ as well as that of V^4+^ 2p_3/2_, respectively^43^. The XPS results appear consistent with the XRD and Raman observations. Moreover, the surface elemental composition of different catalysts was estimated, and the data were presented in Table S1 (Supplementary Information). The measured surface P/V ratios are higher than the nominal value, owing to the common P enrichment on the VPO type catalyst^44–46^. Note also that, the surface P/V ratio of 5%-VPO/MCF and 33%-VPO/MCF is higher than that of unsupported VPO catalyst, indicating that the presence of MCF further intensifies the P enrichment on the MCF supported catalysts. On the other hand, the V^4+^/V^5+^ ratio of unsupported VPO is approximately 0.2, whereas the V^4+^/V^5+^ ratio increases with increasing VPO loading over the supported catalysts. It is understandable since the released NH_3_ from the decomposition of NH_4_(VO_2_)HPO_4_ precursor would be captured by MCF, and the reduction of V^5+^ to V^4+^ is promoted by the adsorbed NH_3_. It is worth pointing out that, the amount of adsorbed NH_3_ on the MCF surface is dependent on the VPO loading (i.e. the NH_4_(VO_2_)HPO_4_ content) as well as the free MCF surface; whereas the reduction of V^5+^ to V^4+^ is governed by the amount of adsorbed NH_3_ and the dispersion state of VPO entity. Apparently, a higher degree of V^5+^ to V^4+^ reduction occurs on a supported VPO with a relatively low VPO loading.

### H_2_-TPR analysis

**Figure 4:**
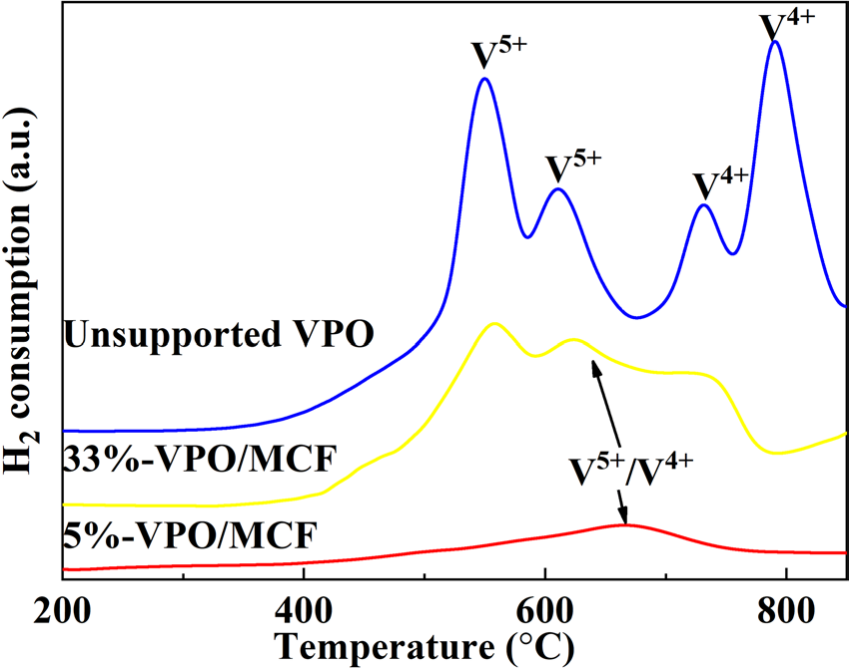
H_2_-TPR profiles of the representative catalysts.

Figure 4 exhibits the H_2_-TPR profiles of different catalysts. The peaks appearing in the 400–650 °C range are ascribable to the V^5+^ reduction, whereas the peaks of 700–850 °C are owing to the V^4+^ reduction^47,48^. Notably, there is only one broad peak in the 400–800 °C range on both 33%-VPO/MCF and 5%-VPO/MCF. The results imply the existence of V^4+^ and V^5+^ species in the two supported catalysts. This agrees with XPS study. On the other hand, there are two peaks in the range of 400–650 °C for the unsupported VPO, attributable to the V^4+^/V^5+^ reduction in δ-VOPO_4_ and β-VOPO_4_, as revealed by the Raman/XPS investigations. The two peaks within 700–850 °C can be owing to the reduction of two kinds of structurally distinct V^4+^ species.

### N_2_ sorption measurement analysis

The N_2_ adsorption-desorption isotherms of different VPO/MCF were measured at 77 K. MCF, 5%-VPO/MCF, and 33%-VPO/MCF exhibit type IV isotherms^49^ with typical H1 type hysteresis loops at the relative pressure of 0.6–1.0 (not shown). The results suggest that the MCF support retains three-dimensional (3D) mesopores even at a VPO loading of 33%^36^. As shown in Table S2 (Supplementary Information), the average pore diameter increases whereas the surface area and pore volume decreases with increasing VPO loading. This is likely due to the existence of VPO components inside the pores and thus the pore-plug as a result. Notably, as the VPO loading is increased, the aggregation of plate-like VPO particles occurs, generating the slit-shaped pores and causing increment in pore diameter.

### Pyridine-adsorption FTIR analysis

The type and relative amount of acid sites has been characterized by FTIR spectroscopy using pyridine as a probe. The Brønsted and Lewis acid sites can be identified in view of the IR bands at ca. 1540/1640 cm^−1^ and 1439/1447/1580/1597 cm^−1^, respectively. The co-presence of B/L acid sites can be confirmed by the IR band of 1490 cm^−1^ ^50^. As shown in Fig. S1, the IR bands at 1439 and 1594 cm^−1^ can be assigned to the Lewis acid sites over the unsupported VPO. Similarly, the IR bands of 1447 and 1595 cm^−1^ over pure MCF, 5%-VPO/MCF, and 33%-VPO/MCF are attributable to the L acid sites^50–53^. Notably, only Lewis acid sites essentially exist on the MCF support and this L-type acid site has little contribution to the target reaction^54^. Both B and L acid sites co-exist over 5%-VPO/MCF, 33%-VPO/MCF, and unsupported VPO, but the fraction of B acid sites in the overall acid sites gradually decreases with increasing VPO content. Interestingly, the nature of L acid sites over MCF support and VPO entity is distinct in view of their IR band shift; in other words, co-existence of B and L acid sites on the VPO entity could account for the observed catalytic activity, whereas the variation in the ratio of B/L acid sites is also responsible for the distinct catalytic behavior of supported/unsupported VPOs.

### NH_3_-/CO_2_-TPD analysis

**Figure 5:**
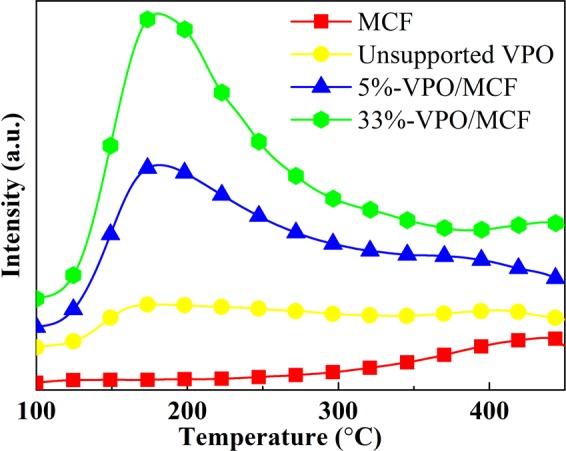
NH_3_-TPD profiles of the catalysts.

The catalyst acid/base characters were further investigated using NH_3_-/CO_2_-TPD and the results are shown in Figs. 5 and S2 and Table S3 (Supplementary Information). As shown in Fig. 5, the NH_3_ desorption peaks over 5%-VPO/MCF and 33%-VPO/MCF emerges at 150–275 °C, suggesting the presence of weak acid sites on the MCF supported VPO catalysts^29^. On the unsupported VPO, the NH_3_ desorption peaks appear at 150–300 °C and 350–450 °C, indicating the existence of weak and medium strong acid sites. Over pure MCF, no peak observed in the range of 150–300 °C, suggesting that the weak acid sites presenting on 5%-VPO/MCF and 33%-VPO/MCF are not originated from MCF^31^. Obviously, the quantity of acid sites on 5%-VPO/MCF and 33%-VPO/MCF is remarkably greater than that of the unsupported counterpart, and the acidity also increases with increasing VPO loading. The application of MCF not only changes the strength but also enhances the quantity of acid sites. The reason for that change in surface acidity is mainly due to the variation in surface V^4+^/V^5+^ ratio as revealed by XPS and H_2_-TPR investigations. Notably, there is no NH_3_ desorption peak over MCF in the 300–450 °C range, indicating NH_3_ can rather strongly adsorb on the MCF support. Be aware of the fact that during the activation of the MCF supported VPOs, accompanying with the decomposition of the NH_4_(VO_2_)HPO_4_ precursor, the released NH_3_ can be resident in fairly high concentration on the MCF surface, which in turn modifies the surface V^4+^/V^5+^ ratio via a NH_3_-induced reduction of V^5+^ to V^4+^.

As Fig. S2 (Supplementary Information) showed, the CO_2_ desorption peaks over the pure VPO and MCF centered at around 450 °C, indicating that the strong basic sites exist on the unsupported VPO as well as the MCF support. Over 5%-VPO/MCF, two CO_2_ desorption peaks at 241 and 446 °C can be observed; whereas over 33%-VPO/MCF, two CO_2_ desorption peaks at 198 and 421 °C can be found, indicating that both supported catalysts have weak and strong basic sites. Clearly, the introduction of MCF changes not only surface acidity but surface basicity as well. According to the previous study^31^, the basicity of VPO can be significantly affected by the P/V ratio. As revealed by the XPS results in this study, 33% VPO/MCF shows the highest surface P/V ratio. In line with the CO_2_-TPD profiles over 33%-VPO/MCF, it is reasonable to deduce that a higher P/V ratio corresponds to a higher density of weak basic sites. If the catalyst acidity and basicity is considered together to correlate with the catalyst performance; clearly, higher concentration of weak acidic and basic sites are responsible for better catalytic activity.

### Catalytic performance

#### Effect of VPO loading

**Figure 6:**
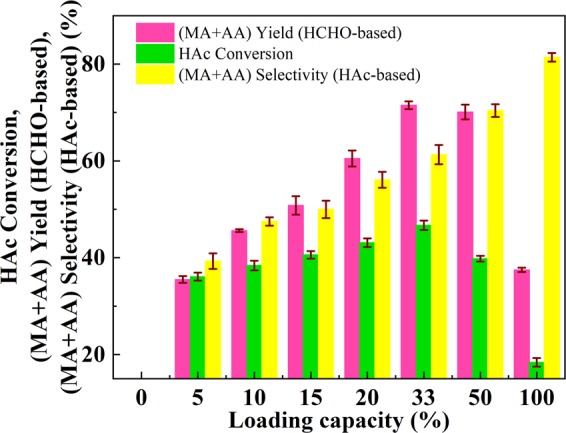
Effect of VPO loading on catalyst performance. T = 360 °C, HAc/HCHO = 2.5/1 (*n*/*n*), carrier flow rate = 40 mL/min, oxygen concentration = 2.25 vol.%, LHSV = 0.44 mL·h^−1^·g_cat_^−1^.

A typical condensation of formaldehyde with acetic acid catalyzed by VPO/MCF with various VPO loading was investigated at atmospheric pressure, a mixture of acetic acid and formaldehyde (15.25 mmol to 6.1 mmol) was fed into the reactor (1.33 mL/h). The reaction temperature was 360 °C and the pre-mixed N_2_ and air (total flow rate = 40 mL/min, and the oxygen flow rate is 0.9 mL/min) was used as a carrier. The MCF supported VPO catalysts with the VPO loadings of 10, 15, 20, and 50% were also prepared and evaluated to identify the optimum VPO content. As shown in Fig. 6, the MCF itself (loading capacity = 0%) shows no catalytic activity under the applied conditions and the (AA + MA) selectivity (HAc-based) was kept rising as the loading capacity increased to 100% (pure VPO), indicating that the activity was essentially originated from the VPO entities. In addition, the yield of (AA + MA) (HCHO-based) and HAc conversion increased notably with increasing VPO loading from 5% till 33% and then decreased with further increasing VPO loading (50%), suggesting that an optimal VPO loading is 33% at which the maximum VPO dispersion is achieved. According to our previous studies^16,20,26^, it has been verified that the surface acid-base property is critical to determine the catalytic performance of the current reaction. And according to the characterization results (see below), it is known that the presence of MCF together with VPO loading shows a significant influence on the surface P/V and V^4+^/V^5+^ ratios, which in turn modifies the surface acidity and basicity of catalyst accordingly.

#### Effect of HCHO/HAc feedstock

The production of AA (MA) through the condensation of HCHO with HAc has received substantial interest from both academic and industrial communities. Formalin^20,26^, trioxymethylene^55^, and dimsethoxymethane^56^ were employed as formaldehyde source. As shown in Fig. S3 (Supplementary Information), a mixture of acetic acid and formaldehyde (1 mmol trioxymethylene is equivalent to 3 mmol formaldehyde, and 1 mmol dimsethoxymethane is equivalent to 1 mmol formaldehyde) with a ratio of 15.25 mmol to 6.1 mmol was fed into the reactor. The reaction temperature is 360 °C and the total carrier flow rate is 40 mL/min (2.25 vol. % O_2_ in N_2_). Clearly, both HCHO-based (AA + MA) yield (71.5%) and HAc-based (AA + MA) selectivity (61.3%) were found to be higher when formalin was used as FA source. According to our previous studies, the presence of water and methanol (as a stabilizing agent) in formalin was beneficial for (AA + MA) production. In cause dimethoxymethane was used as FA source, the highest HAc conversion (64.3%) was obtained but the (AA + MA) selectivity (HAc-based) was the lowest (29.5%). A lot of methanol was formed as dimethoxymethane was fed, and plentiful methyl acetate was generated. The influence of HAc/HCHO ratio on catalytic performance was studied (Supplementary Information, Fig. S4). Notably, the catalytic activity was dependent on the ratio of HAc to HCHO in the feedstock. The yield of (AA + MA) (HCHO-based) and (AA + MA) selectivity (HAc-based) increased significantly with increasing HAc/HCHO ratio till 3/1 and then nearly maintained with further increasing HAc/HCHO ratio, indicating a maximum condensation between HAc and HCHO can be achieved at a HAc/HCHO ratio being 3 over the current catalyst. On the other hand, the HAc conversion decreased continuously with increasing HAc/HCHO ratio till 4/1. Therefore, a fixed HAc/HCHO ratio of 3/1 was adopted in the following studies.

#### Effect of operating parameters

The influence of carrier flow rate, oxygen concentration, and liquid hourly space velocity (LHSV) on catalyst performance was investigated over 33%-VPO/MCF and the results are shown in Figs. S5 and S6 (Supplementary Information), and 7, respectively. The data in Fig. S5 (Supplementary Information) demonstrate that the carrier flow rate could have a direct impact on the activity: the (AA + MA) selectivity (HAc-based) increases while the HAc conversion decreases continuously with increasing carrier flowrate, and the highest (AA + MA) yield (HCHO-based) and (AA + MA) formation rate was achievable (ca. 64.7% and 42.9 µmol·g_VPO_^−1^·min^−1^) when the N_2_ flowrate raises from 20 to 40 mL/min. Longer contact time would favor deeper conversion of the target products, whereas short contact time could lead to quick release of active sites and accelerate reaction cycles^45^.

Furthermore, the gaseous oxygen content in the feed shows a critical impact on the reaction: it particularly tunes the oxidation state of surface V species and thus the surface acidity and the overall catalyst activity and durability as a result. As shown in Fig. S6, the HAc conversion increases while the (AA + MA) selectivity (HAc-based) decreases continuously with increasing co-fed oxygen concentration. At a co-fed oxygen concentration being 4.5% by volume, the (AA + MA) yield (HCHO-based) and (AA + MA) formation rate turned to be 83.7% and 55.5 µmol·g_VPO_^−1^·min^−1^, respectively. The higher the concentration of oxygen in the feed, the higher the degree of over-oxidation of the desired products as well as HAc.

**Figure 7:**
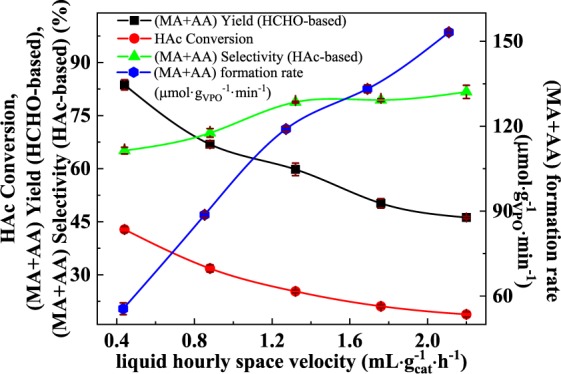
Effect of LHSV on the catalytic performance of 33%-VPO/MCF. T = 360 °C, HAc/HCHO = 3/1 (*n*/*n*), carrier flow rate = 40 mL/min, oxygen concentration = 4.5 vol.% in the carrier.

The influence of liquid hourly space velocity (LHSV) on catalytic performance was also investigated over 33%-VPO/MCF (Fig. 7). As the LHSV varies from 0.44 to 2.22 mL·h^−1^·g_cat_^−1^, the HCHO-based (AA + MA) yield and HAc conversion decline simultaneously, whereas the (MA + AA) selectivity (HAc-based) and (AA + MA) formation rate increase to 81.7% and 153.2 µmol·g_VPO_^−1^·min^−1^, respectively. Note that increase in LHSV would effectively reduce the relative oxygen concentration in the feed, which in turn suppresses the over-oxidation route.

To further study the catalytic performance for the representative catalysts, the catalysts of 5%-VPO/MCF, 33%-VPO/MCF, 50%-VPO/MCF, and unsupported VPO were evaluated under the optimized conditions (carrier flow rate = 40 mL/min^−1^, T = 360 °C, oxygen concentration = 4.5%). The data of HCHO-based yield, HAc conversion, and selectivity as well as carbon balance were determined over the four representatives, the results were presented in Table S4 (Supplementary Information). Obviously, the (MA + AA) selectivity as well as carbon balance was enhanced with increasing VPO loading. However, the HAc conversion and (MA + AA) yield first increases with increasing VPO loading up to 33% and then declines with further increasing VPO content. According to the XPS and NH_3_-TPD investigations, the acid site density on catalyst surface mainly affect the HAc conversion, and an optimized VPO loading could result in a maximized acid site density through the auto regulation of V^4+^/V^5+^ ratio.

To further understand the effect of textural property of support on catalytic activity, other two mesoporous silica, i.e., MCM-41 and SBA-15, were used to prepare the 33%-VPO/MCM-41 and 33%-VPO/SBA-15 catalysts, and the detailed results are shown in Table S5. 33%-VPO/SBA-15 outperforms 33%-VPO/MCM-41, but both are inferior to 33%-VPO/MCF. In terms of the textural/framework structure features of the three distinct meso-structured materials, the MCF is 3D porous, comprising uniform spherical cells interconnected by windows with a narrow size distribution. MCM-41 and SBA-15 are 2D ordered mesoporous material. The pore diameter of MCM-41 and SBA-15 is 3–5 nm and 6–11 nm, respectively; whereas the pore diameter of MCF could be up to 50 nm^36^. The MCF provides more open porous network, allowing accommodation of more VPO guest species, favorable for better molecule accessibility and enhanced mass transfer in the catalyst^35,36^.

#### Catalyst durability

The durability of 33%-VPO/MCF was explored at 360 °C for approximately 140 h, with the LHSV, carrier flow rate, and oxygen concentration being 0.44 mL·h^−1^·g_cat_^−1^, 40 mL/min, and 4.5%, respectively (Supplementary Information, Fig. S7). Slow deactivation was observed within a time-on-stream of 100 h. After that, the catalyst was treated by an air flow (30 mL·min^−1^) for 5 h at the reaction temperature. The activity was essentially recovered. The regenerated catalyst deactivated similarly within an extended period of 40-h.

To verify the possible changes in surface oxidation state and acid site density of catalyst within the reaction procedure, the catalyst used for different time on stream (5, 25, and 72 h, respectively) was characterized by means of XPS and NH_3_-TPD. The results were summarized in Tables S6–8 (Supplementary Information). Obviously, the (MA + AA) yield (HCHO-based) and HAc conversion decreases gradually. On the other hand, the (MA + AA) selectivity declines insignificantly (the inset, Supplementary Information, Fig. S7), demonstrating that catalyst deactivation would be mostly resulted from conversion other than selectivity drop. As Table S7 showed (Supplementary Information), the V species of low oxidation state emerged with time on stream, indicating that the V^5+^ was gradually reduced to V^4+^ and V^3+^. As Table S8 (Supplementary Information) indicated, the medium strong and total acidity gradually decrease with extended reaction period, but the strong acidity essentially retained. Clearly, variation in the surface V oxidation state mainly accounts for the medium strong acidity, very influential on the catalytic performance of the target reaction^20,37^.

**Scheme 1:**
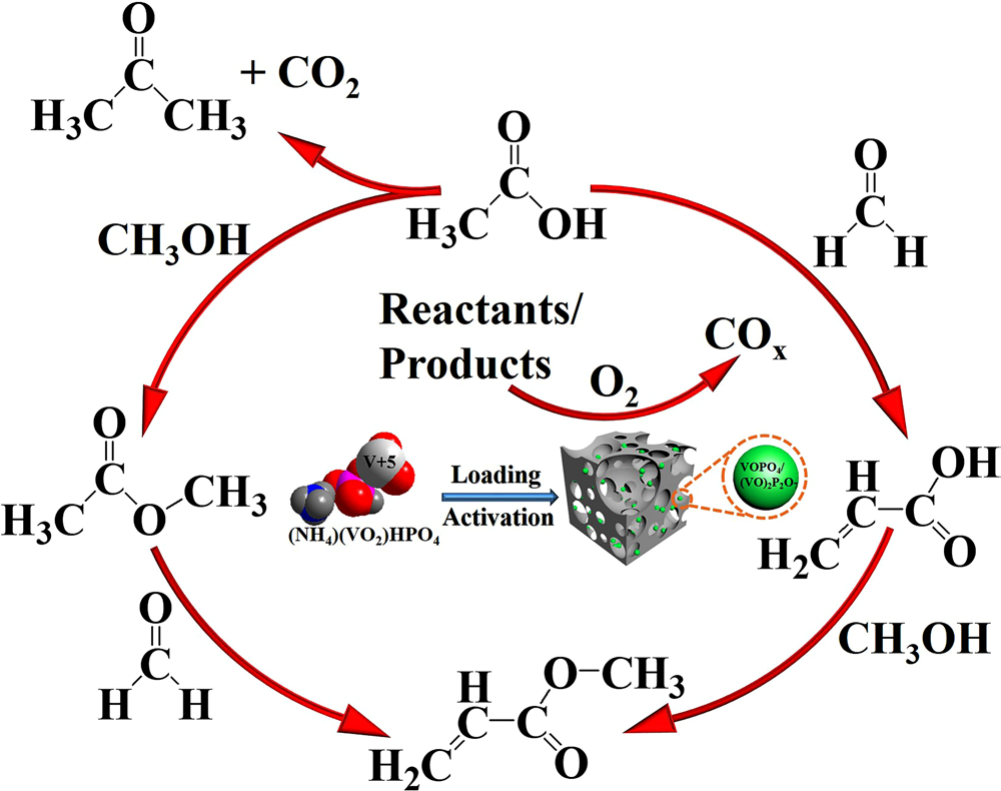
An overview of catalyst design and the involved reaction routes.

Based on the reactants fed and products detected, the involved reaction routes are illustrated in Scheme 1 as well as Eqs. 1–6. HAc reacts with HCHO through nucleophilic addition and dehydration to produce AA (Eq. 1)^16^. This is the main reaction route over the current catalyst system. There are minor side reactions associated with HAc, including bimolecular dehydration and decarboxylation of HAc to acetone and esterification of HAc with methanol to methyl acetate (Eqs. 2 and 3)^31^, the latter can react with HCHO to MA (Eq. 4). MA can also be generated via esterification of AA with methanol (Eq. 5)^28^. CO_*x*_ are produced due to the deep oxidation of reactants/products (Eq. 6), and their contents are sensitive to the oxygen concentration in the feed.

1 $${{\rm{CH}}}_{3}{\rm{COOH}}+{\rm{HCHO}}\to {{\rm{CH}}}_{2}({\rm{OH}}){{\rm{CH}}}_{2}{\rm{COOH}}\to {{\rm{CH}}}_{2}={\rm{CHCOOH}}+{{\rm{H}}}_{2}{\rm{O}}$$

2 $$2{{\rm{CH}}}_{3}{\rm{COOH}}\to {{\rm{CH}}}_{3}{{\rm{COCH}}}_{3}+{{\rm{CO}}}_{2}+{{\rm{H}}}_{2}{\rm{O}}$$

3 $${{\rm{CH}}}_{3}{\rm{COOH}}+{{\rm{CH}}}_{3}{\rm{OH}}\to {{\rm{CH}}}_{3}{{\rm{COOCH}}}_{3}+{{\rm{H}}}_{2}{\rm{O}}$$

4 $${{\rm{CH}}}_{3}{{\rm{COOCH}}}_{3}+{\rm{HCHO}}\to {{\rm{CH}}}_{2}={{\rm{CHCOOCH}}}_{3}+{{\rm{H}}}_{2}{\rm{O}}$$

5 $${{\rm{CH}}}_{2}={\rm{CHCOOH}}+{{\rm{CH}}}_{3}{\rm{OH}}\to {{\rm{CH}}}_{2}={{\rm{CHCOOCH}}}_{3}+{{\rm{H}}}_{2}{\rm{O}}$$

6 $${{\rm{CH}}}_{3}{\rm{COOH}}/{\rm{HCHO}}/{\rm{Products}}+{{\rm{O}}}_{2}\to {{\rm{CO}}}_{x}$$

#### Concluding remarks

The MCF supported vanadium phosphorus oxide with a unique feature of self-regulation of phase constitution and thus surface V^4+^/V^5+^ ratio and acid-base property was technically feasibly fabricated for the first time by employing a specific VPO precursor of NH_4_(VO_2_)HPO_4_ dispersed on MCF. The resulting materials were found to be efficient to catalyze the gas phase HCHO-HAc condensation to AA (MA). The study is the first example to achieve self-regulation of surface V^4+^ and V^5+^ state as well as surface acidity and basicity via simultaneous reduction between the V^5+^ and NH_3_ upon catalyst activation: the released NH_3_ can be captured on the MCF support surface which promotes the redox reaction. Characterizations revealed details about VPO phase structure, surface P/V ratio, surface concentration and oxidation state of V element, reduction behavior of VPO entities, and surface acidity-basicity of VPO/MCFs with respect to the unsupported VPO as well as MCF support. The significant recognition is that both B/L acidity together with weak basicity is critical for forming the desired products. Through simply tuning the VPO loading on MCF, very efficient and selective supported VPO system is achievable: over 33%-VPO/MCF, the (AA + MA) yield of 83.7% (HCHO-based) or a (AA + MA) selectivity of 81.7% (HAc-based) are obtained employing the optimized conditions. This sort of catalyst is relatively durable under the comparatively milder conditions, and in addition, readily reproducible with the help of easy-treatment by air at the identical temperature of reaction.

## Experimental and Methods

### Chemicals

The following chemicals, 1,3,5-trimethylbezene (TMB), Pluronic P123 triblock copolymer (EO_20_PO_70_EO_20_, averaged molecular weight = 5800), tetraethyl orthosilicate (TEOS), ammonium fluoride (NH_4_F), acetic acid (≥99.0%), phosphoric acid (H_3_PO_4_, 85%), ammonium metavanadate (NH_4_VO_3_) were purchased in analytical grade. The commercial HCHO solution (37 wt.%) comprises a small fraction (ca. 6%) of methanol, to stabilize the HCHO component.

### Catalyst preparation

The MCF support was prepared according to the procedures described in literature^57,58^. MCF-supported VPO samples were prepared via a simple impregnation approach which does not employ any organic solvent. NH_4_VO_3_ of 2.34 g was dissolved in 90 mL deionized water, then certain amount of MCF was added at a V/Si atomic ratio of 1/19, 1/2, 1/1 and 1/0, respectively. Being stirred at 90 °C for 6 h, phosphoric acid (85%) with an atomic P/V ratio being 1 was added into the solution. 20 minutes later, the brown mixture was dried at 60 °C under vacuum, further calcined at 400 °C for 16 h in an air flow (60 mL/min).

### Characterization

X-ray powder diffraction (XRD), Raman, BET, XPS, Pyridine-adsorption FTIR, H_2_-TPR, as well as NH_3_/CO_2_-TPD were conducted on the Philips X’Pert MPD Pro X-ray diffractometer, Renishanplc-Reflex Raman spectrometer, ASAP 2020 material physical structure determinator, PHI5000 Versa Probe instrument, Bruker TENSOR 27 spectrometer, and a GC with a TCD detector, respectively. The experimental details were described in SI (Supplementary Information).

### Catalyst evaluation

Catalytic activity was evaluated under atmospheric pressure at 360 °C which was optimized previously. The catalyst amount was 3 g, carrier flow rate was 20–50 mL/min (0–6.8 vol.% O_2_ in N_2_). The mixed HAc and HCHO solution (molar ratio = 1–4) was fed into the reactor and the overall feed rate adjusted in the range of 1.33–6.65 mL·h^−1^ (HCHO feed rate = 6.1–30.5 mmol·h^−1^). The products were analyzed by a GC. The details about experimental set-up as well as the information how to calculate the parameters of *Y*_*AA*__+_*MA*, *X*_*HAc*_, *S*_*AA*__+__*MA*_, *FR*_*AA*__+__*MA*_ and overall carbon balance were provided in SI (Supplementary Information). The experimental error was determined based on the repeated measurements, and indicated in the reaction performance profiles. Generally, the experimental error was found to be less than ±2%.

## Supplementary Information

Supplementary Information
