# Supplementary material for: Vanadium Phosphorus Oxide/Siliceous Mesostructured Cellular Foams: efficient and selective for sustainable acrylic acid production via condensation route
Source: Sci Rep. 2019 Nov 18;9:16988. doi: 10.1038/s41598-019-53180-8 (PMC6861258; doi:10.1038/s41598-019-53180-8)
Supplement: Supplementary file 1 — Supplementary Information [file 41598_2019_53180_MOESM1_ESM.pdf]

Supplementary Information

Vanadium Phosphorus Oxide/Siliceous Mesostructured Cellular Foams system: efficient and selective for sustainable acrylic acid production via condensation route

Jun Liu[1], Peiwen Xu[1], Pengcheng Wang[1], Zhijia Xu[1], Xinzhen Feng[1,*], Weijie Ji[1,*], and Chak-Tong Au[2]

[1] Key Laboratory of Mesoscopic Chemistry, MOE, School of Chemistry and Chemical Engineering, Nanjing University, Nanjing 210023, China

[2] Department of Chemistry, Hong Kong Baptist University, Kowloon Tong, Hong Kong

*Corresponding authors. Tel: +86-25-89686270, Fax: +86-25-89687761,
E-mail: jiwjnju@yahoo.com (W.J. Ji); fxz@nju.edu.cn (X.Z. Feng)

Characterization details.

BET. The samples were degassed at 573 K for 6 h, after which N_2 adsorption was conducted at 77 K.

XPS. The binding energy (BE) was calibrated against the C1s signal (284.6 eV) of contaminant carbon. Elemental surface composition was estimated on the basis of peak areas normalized using Wagner factors. Relative surface concentration of V element with different oxidation state can be estimated through deconvolution analysis of the corresponding XPS peak. For the same batch of sample measured under identical conditions as well as the same parameters adopted for deconvolution analysis, the V^{4+}/V^{5+} ratio of different samples is obtainable for comparison.

H_2-TPR. Hydrogen temperature-programmed reduction (H_2-TPR) was performed from room temperature (RT) to 850 °C at a rate of 10 °C/min in a flow of 5% H_2/Ar (v/v, flow rate = 40 mL/min) and isothermally held at 850 °C until reduction was complete.

NH_3-TPD/CO_2-TPD. Catalyst of 50 mg was first heated in an Ar flow (30 mL/min) to 200 °C and kept at this temperature for 1 h. Then the sample was cooled to 100 °C in the Ar flow. After that, NH_3 or CO_2 adsorption was performed at 100 °C for 1 h. Finally, NH_3(CO_2)-TPD was carried out in an Ar flow (30 mL/min) with the sample being heated to 450 °C at a rate of 10 °C/min. The amount of desorbed NH_3 (in μmol/g) was determined by a titration, in which a HCl solution (0.01 mol/L) was used to absorb the released NH_3. A NaOH solution (0.01 mol/L) was used as the titrant. And the calcium oxalate was used as a Standard to calculated the amount of desorbed CO_2 (in μmol/g).

Pyridine-adsorption FTIR. Bruker TENSOR 27 spectrometer equipped with a cell was used to record the spectra. The samples were pressed into a self-supporting plate (5 mg, 13 mm diameter) and thermally treated at 200 °C under vacuum for 30 min. The pyridine vapor (20 Pa) was dosed into the IR cell at RT and lasted 50 min to reach equilibrium. Then, the cell was vacuumed for 10 min and the spectrum was recorded at 200 °C under vacuum.

Catalyst evaluation details.

All the catalyst powders were pressed, crushed, and sieved to 20–40 mesh for activity evaluation. Two reactors were used for catalyst evaluation, one has an ID of 18 mm without a thermocouple jacket, and the other has an ID of 20 mm with a thermocouple jacket whose outside diameter is 3 mm. The reaction data derived from the two reactors were proved to be reproducible. Catalyst of 3 g was charged into the reactor, and the space above the catalyst bed was filled with quartz chips to preheat the in-coming liquid. Before feedstock introduction, the sample was heated up in a flow of N_2 (30 mL/min) to a desired temperature at a rate of 10 °C/min and kept at this temperature for 2.5 h. When a mixed solution of HAc and HCHO (molar ratio = 1-4) was fed, a mixture of N_2 and air was served as carrier gas. The overall liquid feed rate was 1.33~6.65 mL/h (HCHO feed rate = 6.1-30.5 mmol/h). The products were collected in a cold trap. After 2.5-h reaction, the collected liquid sample was analyzed using a gas chromatograph equipped with a flame ion detector (FID) and a HP-FFAP capillary column (0.32 mm × 25 m). Valeric acid and iso-butyl alcohol were used as internal standards for component quantification. All the catalysts

were first evaluated by screening their performances in terms of the (AA + MA) formation rate in the collected liquid sample based on acetic acid input. Further evaluations were made on a few representative catalysts. In these circumstances, the off-gas was on-line analyzed by a GC equipped with TCD and TDX-01 packed column. It is worth noting that the formaldehyde component cannot be measured by GC analysis, therefore, the formaldehyde conversion cannot be directly determined by using the GC analysis data. In some cases, the unreacted HCHO content was analyzed by the iodometry method. Note that HAc is usually fed significantly excessive in amount over HCHO (in the current study the molar HAc/HCHO is 3:1) to obtain an overall better performance, the by-products such as acetone and CO_x are mainly originated from HAc, thus the data associated with HAc conversion and particularly (AA + MA) selectivity based on the converted HAc is informative and meaningful to evaluate process economy. Because of greater excessive of HAc over HCHO, and the by-products are mainly originated from HAc, the (AA+MA) selectivity based on the converted HAc could be lower than the (AA+MA) yield based on the HCHO input.

Yield of AA+MA (Y_{AA+MA}) based on HCHO is defined by:

$Y_{AA+MA} = n_{(AA+MA)equ}/n_0 \times 100\%$

Where $n_{(AA+MA)equ}$ is the molar quantity of HCHO equivalent to (AA+MA), n_0 is the molar quantity of HCHO fed into the reactor.

Conversion of HAc (X_{HAc}) is defined by the following equation:

$X_{HAc} = (n_0\text{-}n_{measured})/n_0 \times 100\%$

Where n_0 is the molar quantity of acetic acid fed into the reactor, $n_{measured}$ is the mole quantity of unreacted acetic acid.

Selectivity of (AA+MA) (S_{AA+MA}) is defined by:

$S_{AA+MA} = n_{(AA+MA)equ}/(n_0\text{-}n_{measured}) \times 100\%$

Where $n_{(AA+MA)equ}$ is the molar quantity of acetic acid equivalent to (AA+MA), n_0 is the molar quantity of acetic acid fed into the reactor, $n_{measured}$ is the molar quantity of unreacted acetic acid.

The formation rates of AA + MA (FR_{AA+MA}) is defined as the quantity of desired products (in μmol) generated on per unit mass of catalyst per minute.

$FR_{AA+MA} = n_{AA+MA}/(m_{VPO} \times t)$

where n_{AA+MA} is the sum of molar quantity of (AA+MA), mVPO is the mass quantity of VPO component in the sample, and t is the reaction time (150 min).

The carbon balance is calculated in terms of the following equation:

CB (2.5-5 h) = ($N_{acetone} \times n_{acetone} + N_{methyl\ acetate} \times n_{methyl\ acetate} + N_{methanol} \times n_{methanol} + N_{methyl\ acrylate} \times n_{methyl\ acrylate} + N_{acetic\ acid} \times n_{acetic\ acid} + N_{acrylic\ acid} \times n_{acrylic\ acid} + N_{formaldehyde} \times n_{formaldehyde} + N_{CO} \times n_{CO} + N_{CO2} \times n_{CO2})_{measured}/(N_{acetic\ acid} \times n_{0(acetic\ acid)} + N_{formaldehyde} \times n_{0(formaldehyde)} + N_{methanol} \times n_{0(methanol)}$)), where N is the number of carbon in a specific molecule, n is the mole quantity of each component measured by GC and titration.

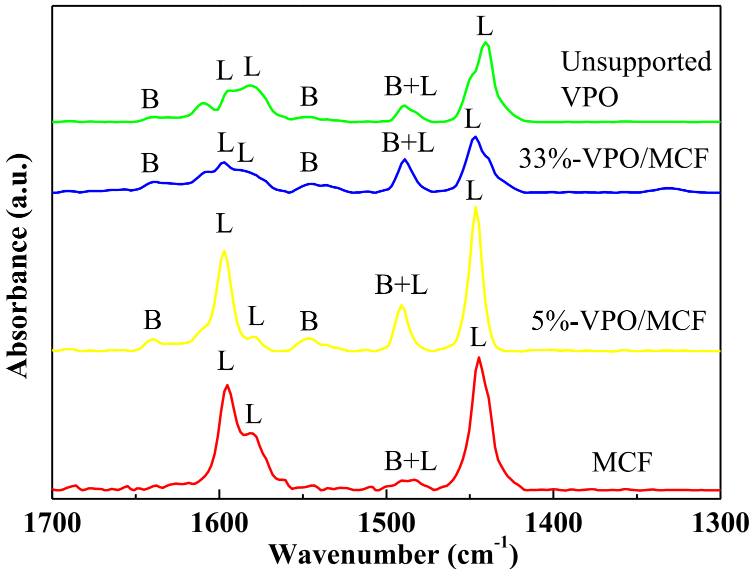

Figure S1. FT-IR spectra of pyridine adsorption on the unsupported and MCF supported catalysts as well as MCF.

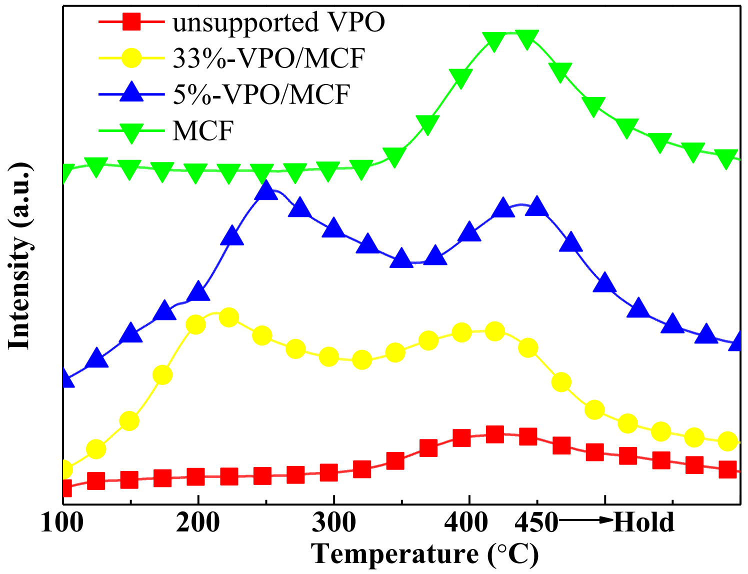

Figure S2. CO_2-TPD profiles of the representative catalysts.

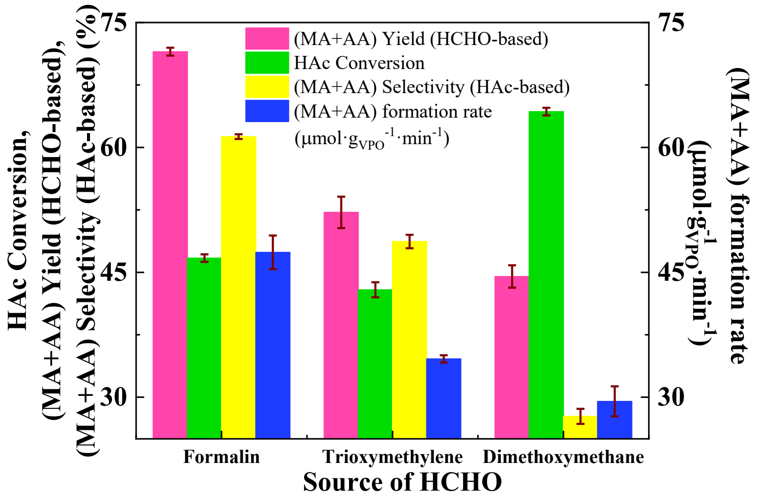

Figure S3. Effect of formaldehyde source on catalytic performance of 33%-VPO/MCF. T = 360 °C, HAc/HCHO = 2.5/1 (*n*/*n*), carrier flow rate = 40 mL/min, oxygen concentration = 2.25 vol. %, LHSV = 0.44 $mL\cdot h^{-1}\cdot g_{cat}^{-1}$.

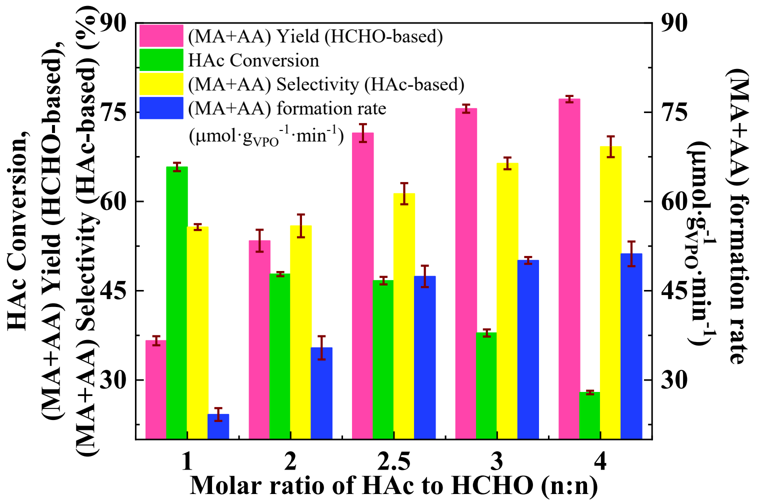

Figure S4. Effect of HAc/HCHO ratio on catalytic performance of 33%-VPO/MCF. T = 360 °C, carrier flow rate = 40 mL/min, oxygen concentration = 2.25 vol. %, LHSV = 0.44 mL·h^{-1}·g$_{cat}^{-1}$.

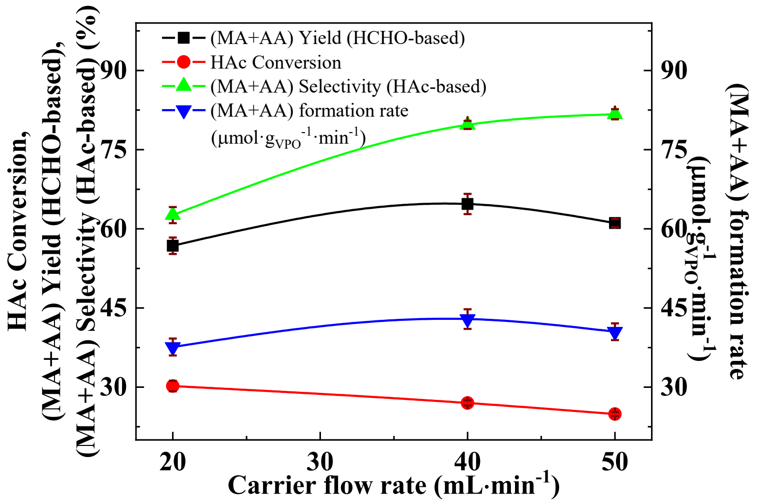

Figure S5. Effect of carrier flow rate on catalytic performance of 33%-VPO/MCF. T = 360 °C, HAc/HCHO = 3/1 (*n*/*n*), oxygen concentration = 0 vol. %, LHSV = 0.44 $mL\cdot h^{-1}\cdot g_{cat}^{-1}$.

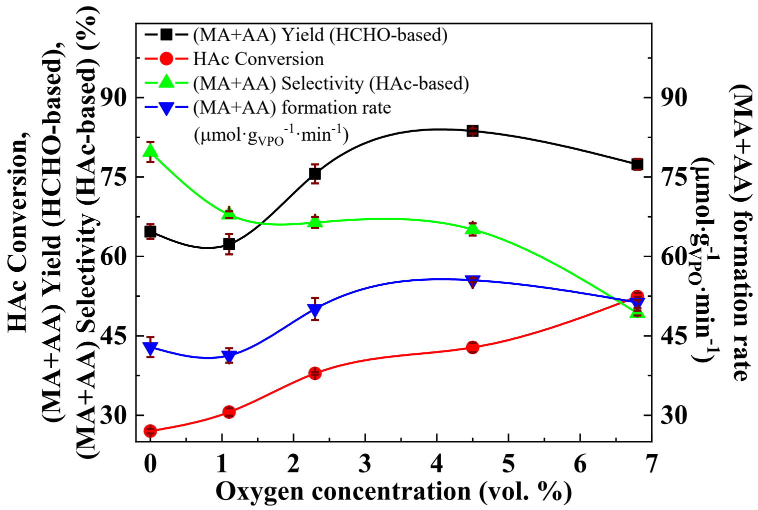

Figure S6. Effect of oxygen concentration on the reaction performance of 33%-VPO/MCF. T = 360 °C, HAc/HCHO = 3/1 (*n*/*n*), carrier flow rate = 40 mL/min, LHSV = 0.44 $mL \cdot h^{-1} \cdot g_{cat}^{-1}$.

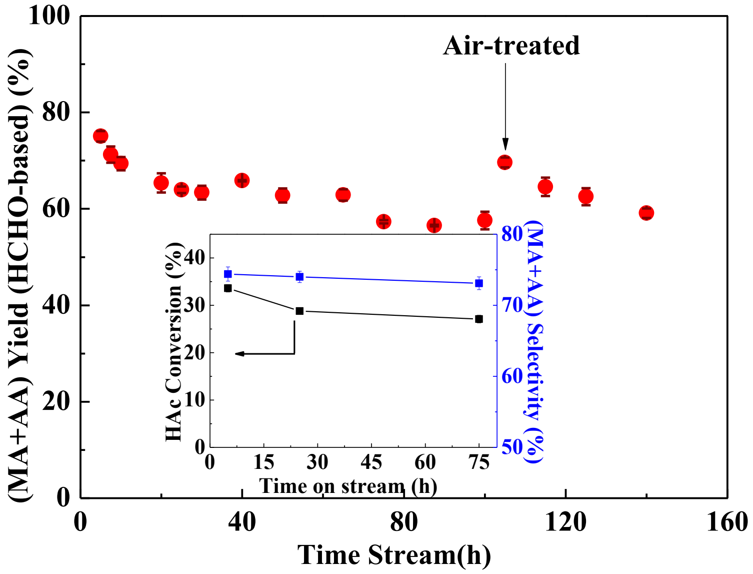

Fig. S7. Durability test over 33 %-VPO/MCF at 360 °C for a period of totally 140 h. At 100 h, the working catalyst was air-treated at 360 °C for 5 h.

Catalyst	Relative surface concentration (at %)					P/V ratio	V^{4+}/V^{5+} ratio
	C1s	Si2p	V2p	P2p	O1s		
**Unsupported VPO**	**24.2**	**/**	**7.8**	**10.7**	**57.3**	**1.4**	**0.2**
**33%-VPO/MCF**	**13.4**	**19.0**	**1.8**	**3.5**	**62.2**	**1.9**	**0.4**
**5%-VPO/MCF**	**9.7**	**23.3**	**0.6**	**1.0**	**65.5**	**1.7**	**0.5**

Table S1. XPS results of the three representative catalysts.

Catalyst	Specific surface area ($m^2 \cdot g^{-1}$)	Pore diameter (nm)	Pore volume ($cm^3 \cdot g^{-1}$)
**MCF**	**542**	**16.5**	**1.87**
**5%-VPO/MCF**	**440**	**9.9**	**1.68**
**33%-VPO/MCF**	**87**	**14.1**	**0.59**
**Unsupported VPO**	**3**	**34.7**	**0.05**

Table S2. Characteristics of MCF, MCF supported and unsupported VPOs.

Catalyst	Total acidity (μmol NH_3/g_{cat})	Basic site distribution (μmol CO_2/g_{cat})		Total basicity (μmol CO_2/g_{cat})
		Weak	Strong	
5%-VPO/MCF	261.9	92.8	96.9	189.7
33%-VPO/MCF	337.8	118.5	95.7	214.2
Unsupported VPO	90.6	/	90.8	90.8

Table S3. The surface acidity/basicity of the VPO catalysts determined by NH_3(CO_2)-TPD.

Catalyst	(MA+AA) yield Based on HCHO (%)	HAc conversion (%)	(MA+AA)* selectivity (%)	(MA+AA) formation rate (μmol·g_{VPO}^{-1}·min^{-1})	Carbon balance (%)
**5%-VPO/MCF**	**52.5±1.4**	**25.7±0.4**	**67.9±1.9**	**147.0±1.9**	**95±0.3**
**33%-VPO/MCF**	**75.0±1.5**	**33.6±0.8**	**74.4±1.1**	**50.1±2.1**	**95±0.6**
**50%-VPO/MCF**	**67.2±1.9**	**30.0±0.9**	**74.8±0.6**	**36.2±1.4**	**96±0.7**
**Unsupported VPO**	**45.8±1.8**	**19.9±0.7**	**76.8±1.0**	**19.0±1.1**	**98±0.5**

Table S4. HAc conversion, selectivity, yield, and carbon balance. All the samples were tested under the following conditions: T = 360 °C, HAc/HCHO = 3/1, carrier flow rate = 40 mL/min, oxygen concentration = 4.5 %, LHSV = 0.44 mL·h^{-1}·g_{cat}^{-1}. The catalytic data were collected during 2.5-5 h reaction. *(AA + MA) selectivity is derived based on the converted HAc.

Catalyst	(MA+AA) yield (HCHO-based) (%)	HAc conversion (%)	(MA+AA)* selectivity (%)	(MA+AA) formation rate ($\mu mol\cdot g_{VPO}^{-1}\cdot min^{-1}$)	Carbon balance (%)
**33%-VPO/MCF**	**75.0±1.5**	**33.6±0.8**	**74.4±1.0**	**50.1±2.1**	**95±0.6**
**33%-VPO/SBA-15**	**61.1±1.0**	**28.7±0.4**	**71.0±1.2**	**40.5±1.4**	**95±1.0**
**33%-VPO/MCM-41**	**49.7±1.3**	**24.4±1.0**	**67.9±1.9**	**32.9±1.0**	**96±0.5**

Table S5. HAc conversion, selectivity, yield, and carbon balance. All samples were tested under the following conditions: T = 360 °C, HAc/HCHO = 3/1, carrier flow rate = 40 mL/min, oxygen concentration = 4.5 %, LHSV = 0.44 $mL\cdot h^{-1}\cdot g_{cat}^{-1}$. The catalytic data were collected during 2.5-5 h reaction. *(AA + MA) selectivity is derived based on the converted HAc.

Catalyst	(MA+AA) yield (HCHO-based) (%)	HAc conversion (%)	(MA+AA)* selectivity (%)	(MA+AA) formation rate (μmol·g_{VPO}^{-1}·min^{-1})	Carbon balance (%)
**Run for 5 h**	**75.0±1.5**	**33.6±0.8**	**74.4±1.0**	**50.1±2.0**	**95±0.6**
**Run for 25 h**	**63.9±1.9**	**28.8±0.5**	**74.0±0.8**	**42.6±1.8**	**97±0.2**
**Run for 72 h**	**59.4±1.4**	**27.1±0.8**	**73.1±0.9**	**39.6±1.0**	**98±0.8**

Table S6. Catalyst performance with time on stream over 33 %-VPO/MCF. T = 360 °C, HAc/HCHO = 3/1, carrier flow rate = 40 mL/min, oxygen concentration = 4.5 %, LHSV = 0.44 mL·h^{-1}·g_{cat}^{-1}. *(AA + MA) selectivity is derived based on the converted HAc.

Catalyst	Relative surface concentration (at %)					P/V atomic ratio	$V^{3+}/V^{4+}/V^{5+}$ atomic ratio
	C1s	Si2p	V2p	P2p	O1s		
Run for 5 h	10.0	19.8	2.2	3.8	64.2	1.7	-/2.4/1
Run for 25 h	28.2	15.7	1.7	3.2	51.1	1.9	0.4/1/-
Run for 72 h	37.7	14.8	1.3	2.3	44.0	1.8	0.6/1/-

Table S7. Estimation of the surface elemental concentration, P/V atomic ratio, and $V^{3+}/V^{4+}/V^{5+}$ atomic ratio of the used 33%-VPO/MCF catalysts subjected to different TOS based on the curve-fitting analysis of the corresponding XPS profiles.

Catalyst	Acid site distribution (μmol NH_3/g_{cat})		Total acidity (μmol NH_3/g_{cat})
	Medium	Strong	
**Run for 5 h**	**118.8**	**140.7**	**259.5**
**Run for 25 h**	**81.1**	**152.6**	**233.7**
**Run for 72 h**	**60.6**	**147.3**	**207.9**

Table S8. Surface acidity of the used 33%-VPO/MCF catalyst subjected to different TOS based on the corresponding NH_3-TPD profiles.
